# Solving the Mystery: A Case Study on Treating a Mandibular First Premolar With Multiple Canals

**DOI:** 10.7759/cureus.63734

**Published:** 2024-07-03

**Authors:** Tikeshwari Gurav, Yogesh Sormare, Aman Thakare, Vikrant Jadhav

**Affiliations:** 1 Conservative Dentistry and Endodontics, Sharad Pawar Dental College and Hospital, Datta Meghe Institute of Higher Education and Research, Wardha, IND; 2 Oral Medicine and Radiology, Sharad Pawar Dental College and Hospital, Datta Meghe Institute of Higher Education and Research, Wardha, IND; 3 Orthodontics and Dentofacial Orthopedics, Sharad Pawar Dental College and Hospital, Datta Meghe Institute of Higher Education and Research, Wardha, IND

**Keywords:** working length determination, obturation master cone, complex root canal morphology, root canal therapy, mandibular first premolar

## Abstract

The mandibular first premolar, which is the anteriormost tooth in the mandibular arch, differs from other teeth in that it typically has two roots and two to four canals. The current case involves treating a mandibular first premolar with two canals through endodontic therapy. The 42-year-old male patient's left mandibular first premolar was diagnosed with irreversible pulpitis. The lateral view X-ray depicted two canals. In the dental procedure, endodontic treatment involves working under an operating microscope and using magnification to treat the affected tooth. After canal shaping the tooth was treated with the crown after it had been cleaned and then adjusted for the bite. As aforementioned, no signs of periapical disease were observed when the tooth was rediscovered a year later. However, this case study indicates that any shift in the anatomy of the canal in premolars should be well monitored and controlled to improve endodontic treatment outcomes of such teeth as the mandibular first premolars.

## Introduction

Since the canal configurations are diverse, knowledge of the teeth’s anatomy and architecture is critical for root canal treatment (RCT). Many anatomical features of teeth need to be understood to perform a successful RCT. The orientation of the working distance is crucial because errors in their detection can result in RCT failure [1,2]. If we have to single out a cause of endodontic failure in individual teeth, inadequate filling of the canals and inadequate instrumentation are the two primary culprits [3]. Several studies have documented anatomical interferences in the root canal systems of mandibular premolars [4]. The rate at which the anatomical features of the first and second mandibular deciduous premolars change varies; that is, they may have one or two extra canals [5,6]. Slowey believes that the "endodontist’s enigma," the 1st premolars in the mandibular region, can often pose a problem to the endodontic treatment [7].

## Case presentation

**Figure 1:**
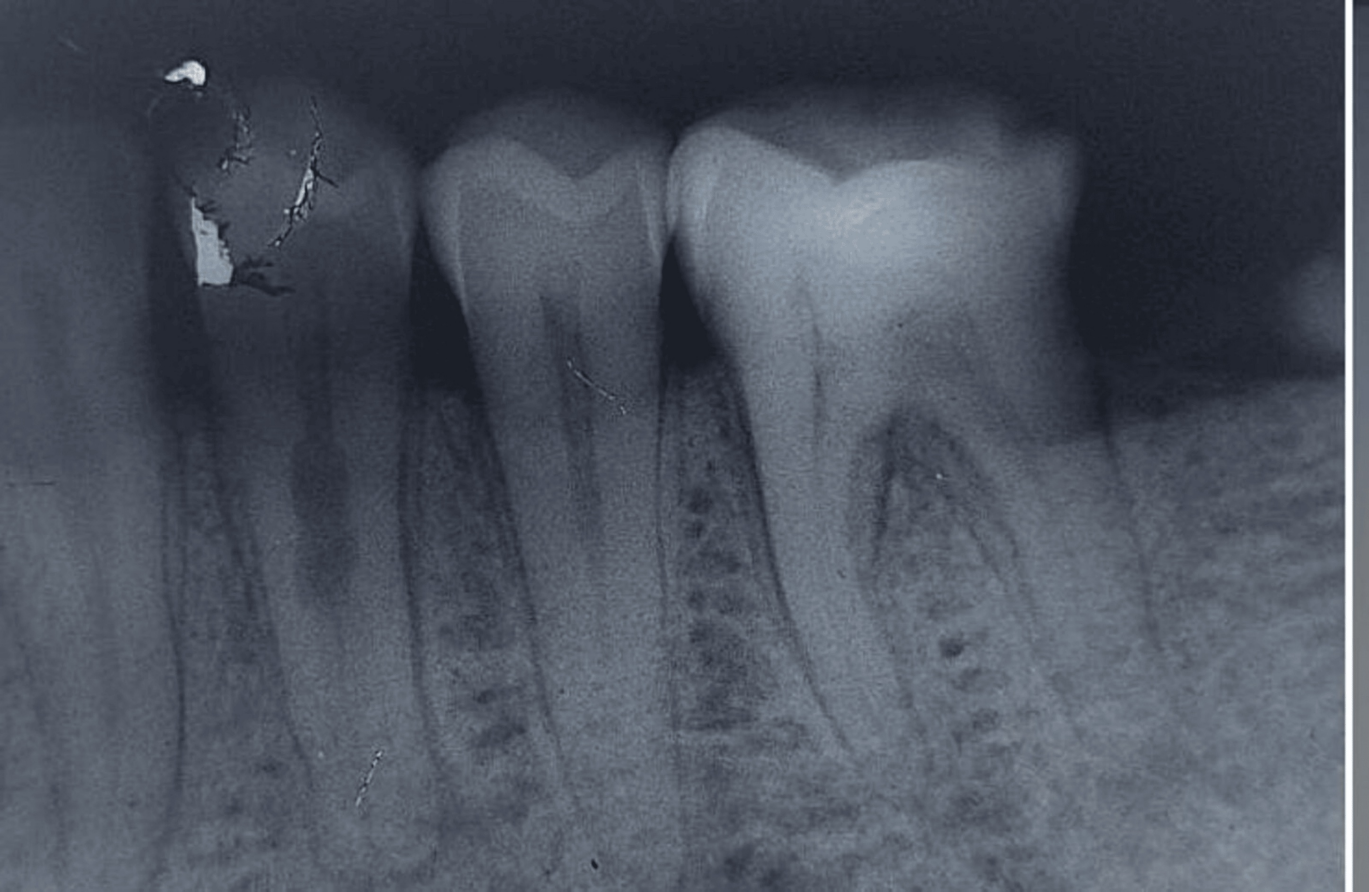
Pre-operative intra-oral periapical radiograph of the mandibular left first premolar in the patient in the case study, showing an unusual anatomy of two roots, and widening of the apical periodontium

A 42-year-old male was referred to Sharad Pawar Dental College and Hospital's Conservative Department for the treatment of the left mandibular first premolar. He had complained of pain for the past 10 days and had a four-month history of being sensitive to cold drinks. Painkillers were used to alleviate it. The patient's medical history was not contributing. The diagnostic tests were performed with the patient's informed permission. Clinical examination showed discomfort on percussion in the left mandibular first premolar. Acute irreversible pulpitis was diagnosed in the tooth after routine clinical tests. Following a radiographic examination, the tooth was discovered to have an unusual anatomy, including two roots, and internal resorption, which indicates periapical pathology and the need for RCT as shown in Figure 1.

Treatment

**Figure 2:**
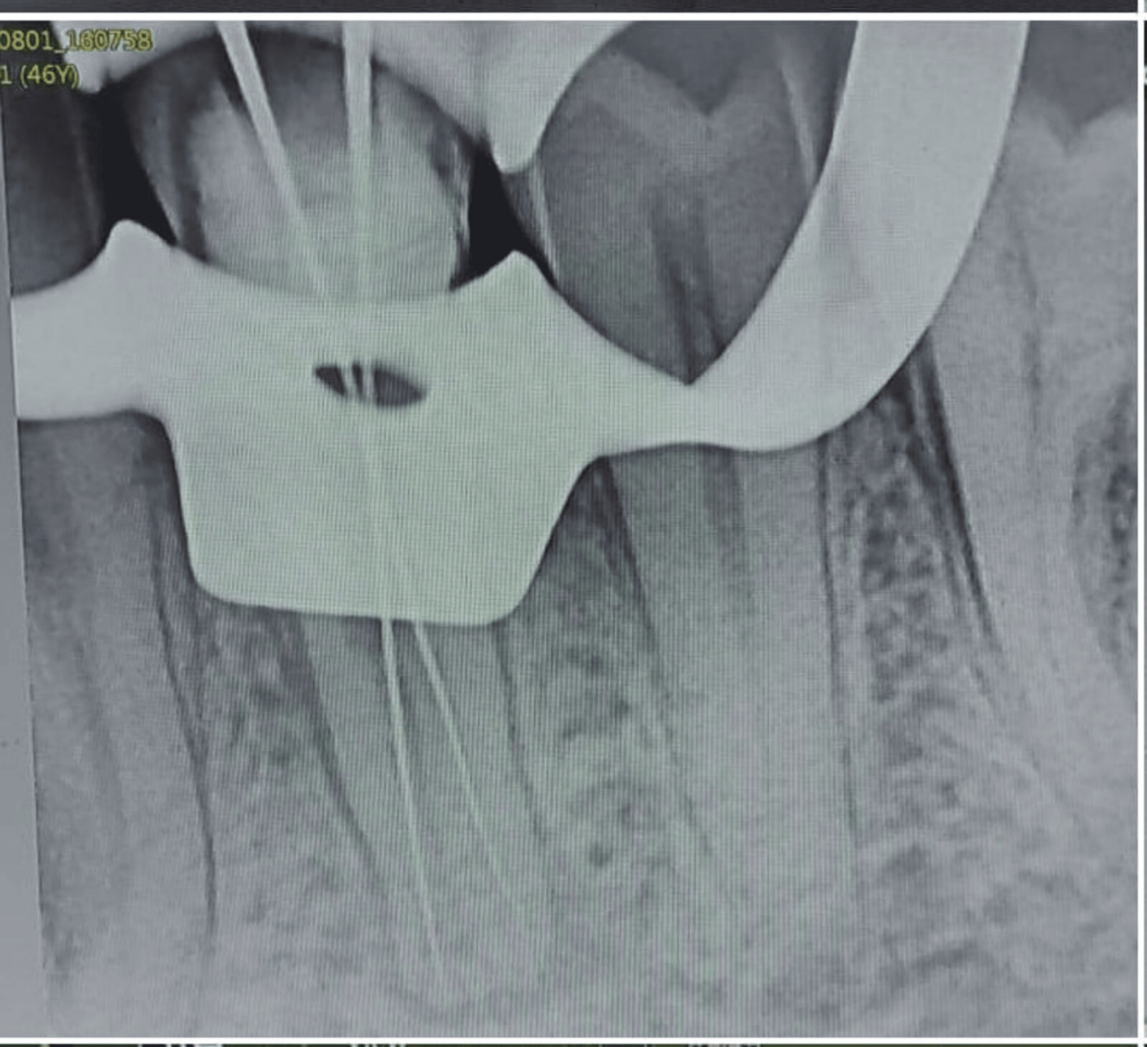
The patency of both canals was confirmed with the #10 K file (Mani Inc., Tochigi, Japan). Subsequently, the initial working length was determined using the #15 K file (Mani Inc., Tochigi, Japan) with the help of Root Zx Mini Apex Locator (J. Morita Corp., USA), which was evaluated using a radiograph

RCT is recommended for a patient, and prior consent was obtained before beginning the procedure. A 2% lignocaine hydrochloride solution with 1:8000 adrenaline was administered via the left inferior-alveolar nerve block to anesthetize the tooth. The rubber-dam isolation made the pulp mandibular premolar with two roots accessible, and the chamber was completed after local anesthesia was administered with due care and preparation. Following the removal of the necrotic pulp tissue, the chamber was cleaned with a 5% sodium hypochlorite solution. Two canal orifices were discovered after thorough inspection, and their integrity was confirmed using a small size K-10. One H-15 and one K-15 were placed in each canal to differentiate between the two roots and canals, using an apex locator the working length was calculated as shown in Figure 2.

Using the gates glidden drills 1, 2, and 3, the orifices have been enlarged and the brush has been brushed straight down from the crown. A separate injection of 17% ethylenediaminetetraacetic acid (EDTA) and 5% sodium hypochlorite was applied to the canals to clean and shape them.

**Figure 3:**
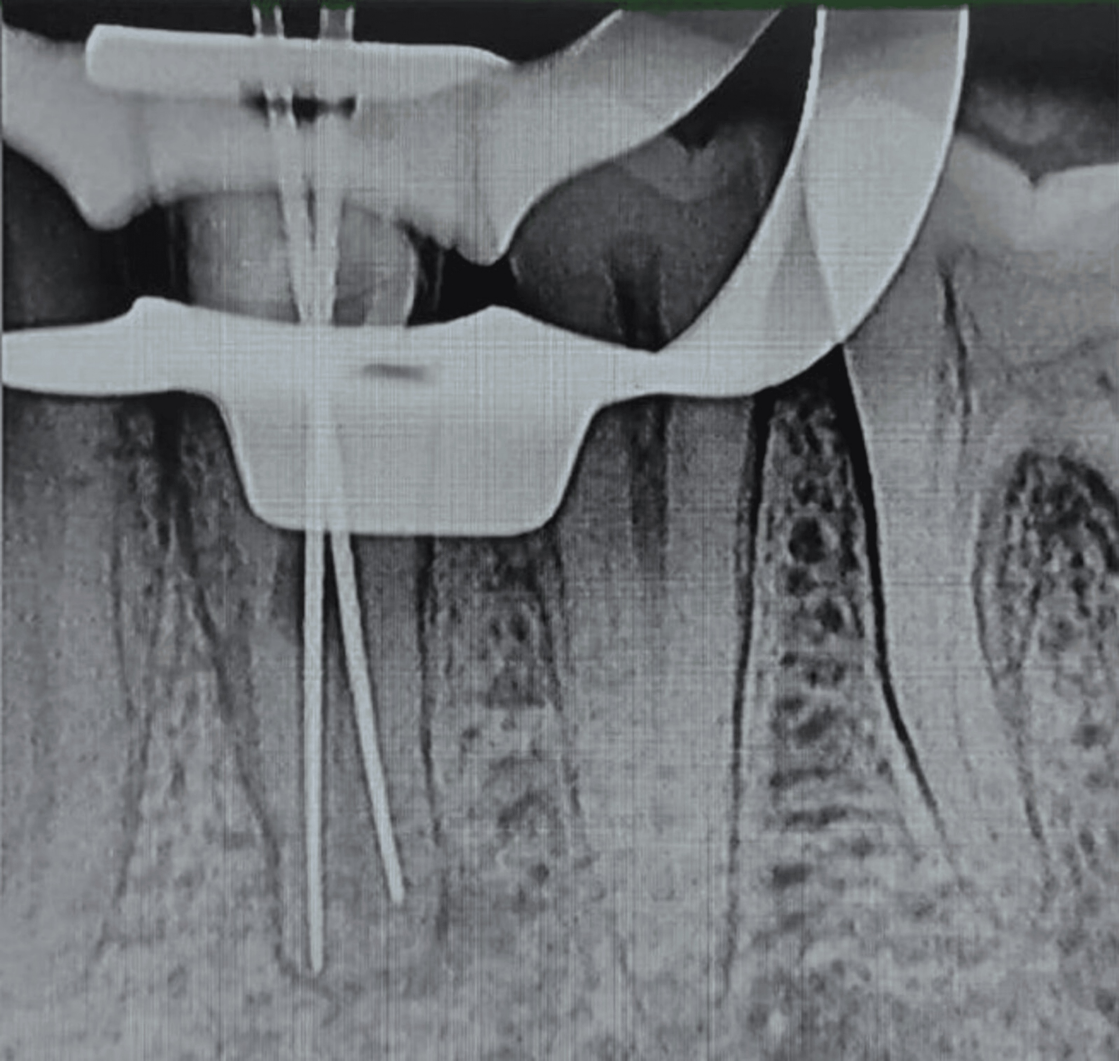
Biomechanical preparation was done using hand-operated files and nickel-titanium rotary instruments (Dentsply Maillefer, Ballaigues, Switzerland). Thorough irrigation with 5.25% NaOCl, normal saline, and 0.2% chlorhexidine was also done simultaneously. The master cone fit was checked and confirmed on the radiograph following the final working length determination

One coronal canal was split in the middle third, indicating the separation of both roots, according to a working-length radiograph. The two canals emerged from their respective roots through distinct apical foramina. Biomechanical preparation requires the use of protaper hand files. After administering calcium hydroxide intracanal medication, the canals were sealed with an intermediate restorative material. The canals were cleaned with Protaper hand files, normal saline, 17% EDTA, and 5.25% sodium hypochlorite at the following appointment. The canals were completely dried and filled with standardized 2% taper protaper Gutta-percha. Following the obturation, the radiograph revealed that the canals were completely sealed, as shown in Figure 3.

**Figure 4:**
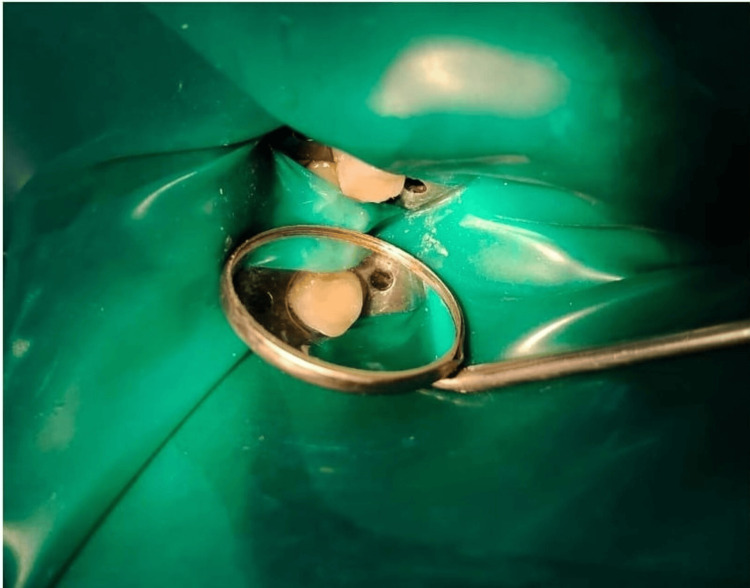
Displays final restoration after one week

A week after the endodontic treatment, the patient was inspected, and the final restoration was put in place as shown in Figure 4.

They were then sent to the prosthodontics department for further care.

## Discussion

Dental professionals frequently think that there is a set quantity of roots and/or canals in every tooth. Despite a complete examination of research materials, tooth morphology deviations from the norm are not uncommon [8]. When treating teeth with endodontic therapy, any anatomical variations, such as additional canals and roots, should be taken into account. The existence of microorganisms in canals that are not regularly cleaned has been linked to treatment failure [9,10]. In one of its studies, the University of Washington looked at the failure rate of non-surgical root canal therapy on all teeth. The first premolar in the mandible has the highest rate (11.45%) [11].

More apical transportation occurs during the preparation of curved oval canals using rotating instruments as opposed to push-pull filing movement [12]. To shape canals with complex curvatures, it is recommended to use NiTi file systems, which have more flexible and less tapered instruments, like K3 and RaCe [13]. The apices of the mandible's second premolar teeth are near the mental foramen and the neurovascular structures that traverse it. This important point needs to be addressed and confirmed in these discussions.

One of the most common reasons for difficulty identifying the second canal is a lack of access, which results in a dentine shelf over the canal. The additional canal frequently exits the primary one at a sharp angle, much like a right angle. Preparing an access cavity is essential for successfully treating all orifices. Cleaning, filling, and shaping teeth can be difficult due to restricted access. Undiagnosed extra roots or canals can lead to treatment complications and endodontic failure. Proper access to the pulp chamber is critical due to its small size and limited visibility in premolars [14].

The best way to picture the canal configuration, according to Slowey, is as a lowercase letter "h," with the main canal representing the straight part of the letter and the second canal located at a sharp angle from the straight canal around the mid root [15]. In a minimum of 23% of the initial mandibular bicuspids, Zillich and Dowson discovered a second or third canal. Canals within the root can split at any point [16]. Vertucci's research revealed that 0.5% had three canals at the apex, 25.5% had two, and 74.0% of mandibular first premolars had just one [17]. Green et al. discovered a 4% occurrence of two roots and two foramina. Identifying extra roots or canals during treatment can help avoid acute flare-ups and endodontic therapy failure [18].

Cone-beam computed tomography (CBCT) management of multiple canals in a mandibular first premolar is a topic of debate for some reasons, which may account for its exclusion from the article you cited. Here are some potential reasons: the cost of a CBCT scan for the patient can also be a consideration, especially if alternative diagnostic tools are available. Avoiding CBCT may be desirable in some patients, particularly when the clinical suspicion of multiple canals can be validated with less exposure. When dealing with complex root canal morphology, some clinical recommendations might not need the use of CBCT in every instance, especially if there are other reliable diagnostic techniques.

The points you raised draw attention to crucial factors to take into account when deciding whether to employ CBCT to manage numerous canals in a mandibular first premolar. These are each point's benefits: the fact that the effective radiation dose from CBCT is less than that of a panoramic X-ray and far less than that of a medical CT scan is one of its benefits. CBCT has revolutionized dent maxillofacial structure imaging by providing three-dimensional precision of hard tissue images at a reasonable cost. When a patient's symptoms are poorly localized and only partial information is provided by clinical and periapical radiographs, CBCT can be utilized to identify periapical illness [19].

## Conclusions

The presence of additional roots or root canals in mandibular premolars, which can happen far more frequently than anticipated, presents an endodontic challenge. To acknowledge and address teeth with different anatomical variations, dentists must understand root canal anatomy, be able to interpret radiographs and modify the standard access activity carefully. Effective root canal treatment necessitates an accurate diagnosis and understanding of the anatomy involved. The reported case emphasizes the importance of clinicians understanding internal root canal anatomy and using modified techniques before and during treatment to avoid future flares.
